# Adult Born Periglomerular Cells of Odorant Receptor Specific Glomeruli

**DOI:** 10.3389/fnana.2018.00026

**Published:** 2018-04-10

**Authors:** Anna-Maria Maier, Heinz Breer, Jörg Strotmann

**Affiliations:** Institute of Physiology, University of Hohenheim, Stuttgart, Germany

**Keywords:** olfactory bulb, adult neurogenesis, periglomerular cells, OR37, glomeruli, BrdU, doublecortin

## Abstract

The OR37 subsystem is characterized by a variety of unique features. The odorant receptors (ORs) of this subfamily are selectively tuned to specific ligands which are supposed to play a role in social communication. OR37 expressing sensory neurons project their axons to a single receptor specific glomerulus per bulb which have been shown to be unusually stable in size and to possess a distinct repertoire of periglomerular cells. Since the neuronal network surrounding glomeruli is typically modified by the integration of adult born neurons, in this study it was investigated whether the number of adult born cells might be different for OR37 glomeruli compared to other OR-specific glomeruli. Towards this goal, 23 days after BrdU injection, BrdU labeled cells in the proximity of OR37A glomeruli as well as around OR18-2 and OR256-17 glomeruli were determined. It was found that the number of BrdU labeled cells in the periglomerular region of OR37A glomeruli was significantly lower compared to glomeruli of the other OR types. This finding was in line with a lower number of neuroblasts visualized by the marker protein doublecortin. Double labeling experiments for BrdU and marker proteins revealed that despite a relatively high number of calretinin expressing cells at the OR37A glomeruli, the number of cells co-stained with BrdU was quite low compared to other glomeruli, which may point to an individual turnover rate of this cell type for different glomeruli. Together, the results of the present study support the notion that the neuronal network at the OR37 glomeruli is less dynamic than that of other glomerulus types. This indicates a specific processing of social information in OR37 glomerular networks.

## Introduction

Odorant receptors (ORs) of the OR37 subsystem display several unique features setting them apart from other ORs. They are exclusively found in mammals and share a high degree of sequence homology, even across species borders (Hoppe et al., 2003, 2006). Recent studies have shown that OR37 sensory neurons are activated by long chain aliphatic aldehydes, which are probably inherently produced by mice and represent untypical odorants due to their low volatility (Bautze et al., 2012, 2014). OR37 ORs are expressed in sensory neurons that are clustered in a restricted patch in the olfactory epithelium and project their axons to a single glomerulus in the ventral part of the olfactory bulb (Strotmann et al., 1999, 2000). This is in contrast to canonical ORs which are typically expressed in OSNs widely distributed throughout the epithelium that project to two or more glomeruli in the olfactory bulb (Mombaerts et al., 1996; Mori and Sakano, 2011). In the olfactory bulb, OSNs synapse onto mitral/tufted cells which project to higher brain centers, mainly the lateral olfactory tract (López-Mascaraque and de Castro, 2002; de Castro, 2009). OR37 projection neurons however have been shown to connect to nuclei in the amygdala and hypothalamus; brain regions typically related to stress and social phenomena (Bader et al., 2012a,b). Thus, the OR37 subsystem is supposed to be involved in social communication and may elicit innate reactions in mice (Klein et al., 2015). Based on this notion, it has been hypothesized that information processing of the OR37 subsystem in the olfactory bulb might require specialized structural features of the underlying neuronal network. This notion is supported by a recent study demonstrating that the size of glomeruli for the OR37A OR is particularly stable (Maier et al., 2017); a feature quite different from the highly variable glomerulus size of other OR types (Bressel et al., 2016). Furthermore, in the periglomerular region of OR37A glomeruli, a distinct repertoire of cell types was found which are characterized by distinct calcium binding proteins (Maier et al., 2017). Periglomerular cells represent a highly heterogeneous population of interneurons which are involved in modulating glomerular activity (Parrish-Aungst et al., 2007; Nagayama et al., 2014; Kosaka and Kosaka, 2016). In fact, the periglomerular network of interneurons is supposed to form microcircuitries for individual glomeruli, which adapt to the processing of various odor qualities (Wachowiak and Shipley, 2006; Linster and Cleland, 2009). The neuronal networks in the bulb, however, are not static but rather highly dynamic, due to the continuous replacement of bulbar interneurons (Alvarez-Buylla and Lim, 2004; Whitman and Greer, 2007; García-González et al., 2016; Grelat et al., 2018). Such adult born interneurons are thought to shape the neuronal network in response to the different odorants which are processed in the bulb (Bastien-Dionne et al., 2010; Sawada et al., 2011). Adult neurogenesis is therefore considered a prerequisite for the plasticity of the network in the olfactory bulb (Alonso et al., 2006; Moreno et al., 2009; Sakamoto et al., 2014; Mandairon et al., 2018). The OR37 subsystem however is thought to be tuned to distinct odorants and exert an inherent function in social communication (Klein et al., 2015). This raises the question whether replacement of the involved interneurons might be of the same relevance for the periglomerular networks of OR37 glomeruli as for glomeruli of other ORs. Therefore, we have investigated the appearance of adult born cells around OR37 glomeruli in comparison to glomeruli of the ORs OR18-2 and OR256-17. Towards this goal, transgenic mice in which glomeruli for different ORs can be visualized by fluorescent markers were injected with the proliferation marker BrdU to label adult born cells in the vicinity of distinct glomeruli.

## Materials and Methods

### Mice

In the present study, adult mice of both sexes which carry an inserted reporter allele at the locus of specific ORs were used to visualize the corresponding glomeruli. Glomeruli of OR37A (Olfr155), OR37B (Olfr156) and OR37C (Olfr157) were analyzed in comparison to glomeruli of OR18-2 (Olfr78 or MOL2.3) and OR256-17 (Olfr15), respectively. For OR37A and OR37C each, two different strains of reporter mice were used which possess an additional IRES-tau-lacZ or an IRES-tau-GFP allele at the respective gene locus. Reporter mice for OR37B carry an additional IRES-tau-lacZ allele at the OR37B gene locus. As glomeruli in the different reporter strains for OR37A and OR37C do not show structural differences except for the reporter protein (Strotmann et al., 2000), data for the lacZ- and GFP-reporter strains were pooled for analyses for each OR. Reporter mice for OR256-17 carry an IRES-tau-GFP sequence at the locus of OR256-17 (Luxenhofer et al., 2008). These strains were bred on a C57BL/6J background (Strotmann et al., 2000; Luxenhofer et al., 2008). Reporter mice for OR18-2 possess an IRES-GFP-IRES-tau-lacZ sequence at the OR18-2 locus and were bred from a CD-1 background (Conzelmann et al., 2000). For analyses of GAD65 expressing interneurons, the named reporter mouse strains for OR37A, OR18-2 and OR256-17, respectively, were crossed with a transgenic mouse strain carrying a GAD65-IRES-tdTomato allele on a C57BL/6J background (Besser et al., 2015). Mice were housed in groups at a 12-h light/dark cycle in the Animal Research Unit of the University of Hohenheim and had access to food and water *ad libitum*. Experiments were carried out in accordance with the Council Directive 2010/63EU of the European Parliament and the Council of 22 September 2010 on the protection of animals used for scientific purposes (T126/14 Phy).

### BrdU Injections

Mice were injected on four consecutive days, three times a day. Fifty milligram per kilogram BrdU in 0.9% sterile saline were administered intraperitoneally in each injection, according to the bodyweight of the individual. Mice were sacrificed either 12, 19, 23 or 26 days after the last BrdU injection. Until sacrifice mice were kept under standard housing conditions as described above.

### Tissue Preparation

Mice were sacrificed by cervical dislocation and subsequent decapitation. All bones of the head surrounding the brain and the nasal turbinates were removed. No decalcification protocol was applied for the remaining bone parts. The resulting tissue samples were fixed in 4% paraformaldehyde at 4°C for 24 h and cryoprotected by immersion in 25% sucrose in phosphate buffered saline (PBS) at 4°C overnight. Subsequently, brains were embedded in tissue freezing medium on dry ice and kept at −20°C until cryosectioning. Coronal cryosections of 12 μm thickness were used for immunohistochemistry analyses.

### Immunohistochemistry

For the lacZ reporter strains, a pre-staining was performed before the BrdU immunohistochemistry protocol. The tissue sections were washed for 10 min in PBS and incubated with an antibody against ß-galactosidase in 10% normal goat serum and 0.3% Triton in PBS overnight at 4°C. After primary antibody incubation, slides were washed three times in PBS and incubated with a corresponding secondary antibody for 2 h at room temperature. Subsequently, sections were washed again three times in PBS and the resulting staining was fixed in 4% paraformaldehyde 1 h at 4°C. Slides were then passed over to the BrdU immunohistochemistry protocol.

For BrdU immunohistochemistry experiments, tissue sections were washed three times in TRIS buffered saline (TBS) and subsequently transferred to 2 N HCl where they were incubated for 30 min at 37°C to denature DNA. Afterwards tissue sections got neutralized in sodium borate buffer (pH 8.5) for 10 min at room temperature. After washing again three times in TBS, tissue sections were blocked for 30 min with 1% BSA and 0.3% Triton in TBS and incubated with the respective primary antibodies (see Table 1) in the same blocking solution overnight at 4°C. For co-staining experiments, both antibodies were applied simultaneously. A specific antibody against BrdU was applied together with an antibody against DCX, NeuN, TH, CB or CR, respectively. A detailed list of all deployed antibodies is given in Table 1. After primary antibody incubation, sections were washed again three times in TBS and blocked for 15 min before incubating with the corresponding secondary antibodies, using the same blocking solution. Finally, sections were washed again three times before DAPI staining (dilution 1:1000 in TBS) was conducted and stopped in bidest. Slides were mounted using Mowiol.

**Table 1:** Antibodies.

	Code	Supplier	Dilution	Host
**Primary antibodies**
BrdU	MCA2060	Bio-Rad	1:300	Rat
Calbindin	sc-7691	Santa Cruz	1:300	Goat
Calretinin	sc-11644	Santa Cruz	1:300	Goat
Doublecortin	AB2253	Millipore	1:1000	Guinea pig
NeuN	ab177487	Abcam	1:1000	Rabbit
Tyrosine Hydroxylase	ab112	Abcam	1:700	Rabbit
β-Galactosidase	Z3781	Promega	1:1000	Mouse
**Secondary antibodies**
Anti-goat Alexa Fluor 568	A-11057	Invitrogen	1:500	Donkey
Anti-guinea pig Cy3	ab102370	Abcam	1:500	Goat
Anti-mouse Alexa Fluor 488	A-11017	Invitrogen	1:2000	Goat
Anti-rabbit Alexa Fluor 568	ab175694	Abcam	1:500	Donkey
Anti-rat Alexa Fluor 647	ab150155	Abcam	1:500	Donkey
Anti-rat Cy3	AP189C	Millipore	1:500	Donkey

*List of primary and secondary antibodies used in immunohistochemistry experiments in alphabetical order*.

### Image Analyses

Fluorescence multi-channel images were captured using a Zeiss Axioskop 2 plus with an AxioCam MRm camera and processed with ZEN 2009 and Axiovision SE64 4.9 software (Zeiss). For each glomerulus analyzed, a complete series of sections throughout the entire structure was captured. On each image, the region of interest around the regarding glomerulus was outlined in multi-channel images, with a maximum range of four rows of DAPI stained nuclei. This also represents the perimeter in which activated cells can be detected after a ligand-specific stimulation and represents a well-established counting method (Oliva et al., 2008; Bautze et al., 2012, 2014). This outline was marked in a multi-channel image and thereby transferred to marker labeled channels. Marker positive cells were only counted within this outline. BrdU and doublecortin signals, as well as the analysis of colocalization with marker signals, were counted manually in the Axiovision program as the numbers were quite low and the program allowed reassuring colocalization with DAPI and marker signals. Cell countings for NeuN, GAD65, CR and CB were performed on exported Axiovision images using the ITCN tool for nuclear stainings in the ImageJ software. Counted values of each section were summed up for individual glomeruli.

### Statistics

Statistical analyses were performed in SAS 9.4 software generated by SAS Institute from Cary, North Carolina (USA). Generalized linear mixed models with fixed effects for OR types and random effects for animal and glomerulus were applied for BrdU and doublecortin data analysis; with *post hoc* *t*-tests on Least Square Means for the three groups. For absolute numbers as depicted in Figure 4, unpaired *t*-tests were applied. Pearson correlation was applied for the correlation analysis; data for the three OR specific glomerulus types were pooled as no correlation was observed for split groups neither. In all experiments, *n* refers to the number of glomeruli analyzed in the regarding experimental group. The glomeruli of at least three individuals were analyzed for each experimental group. For detailed information on number of deployed animals, see Table 2.

**Figure 1:**
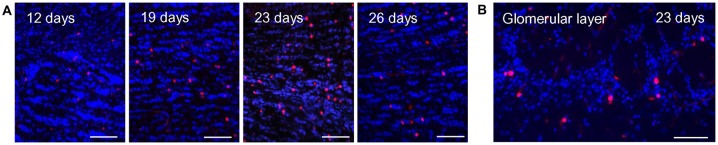
BrdU labeling in the olfactory bulb at different time points after injection. **(A)** BrdU immunoreactive cells (red) and DAPI staining (blue) in the granule cell layer of the olfactory bulb at different time intervals after BrdU injections. The number of BrdU signals visible in the granule cell layer increases from day 12 to 23 days after the last injection and declines again at day 26. Scale bar: 100 μm.** (B)** At 23 days after the last injection, a substantial number of BrdU immunoreactive cells (red) was also visible in the glomerular layer. DAPI staining is shown in blue. Scale bar: 100 μm.

**Figure 2:**
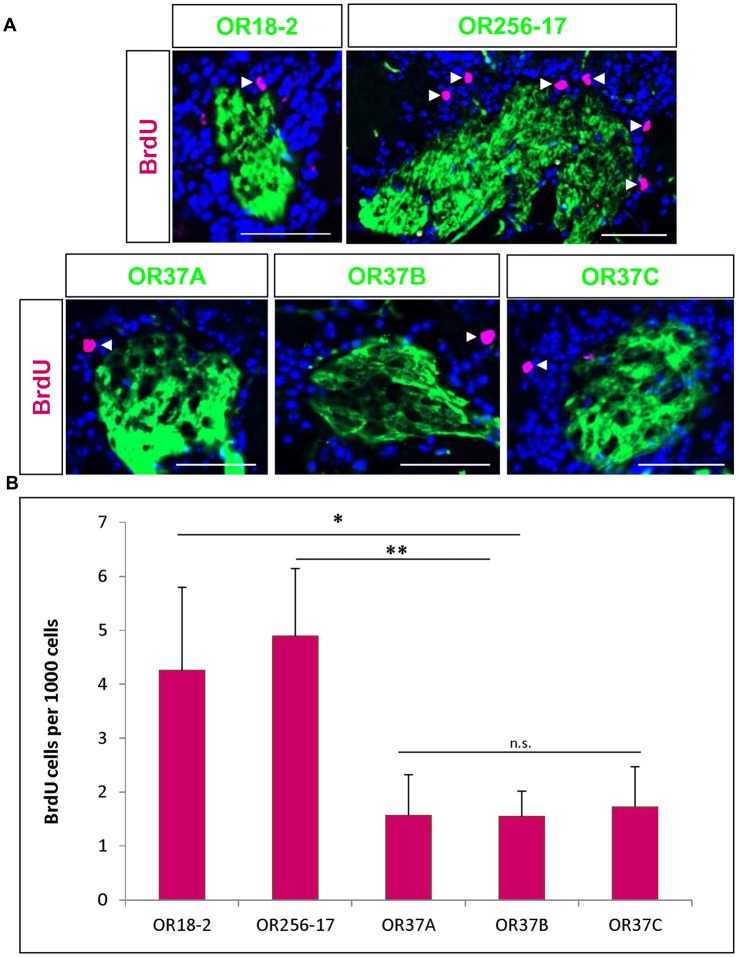
BrdU positive cells in the vicinity of odorant receptor (OR) specific glomeruli. **(A)** Representative images of glomeruli for OR18-2, OR256-17, OR37A, OR37B and OR37C, respectively (green), with BrdU immunoreactive cells (magenta) and DAPI staining (blue). Scale bar: 50 μm.** (B)** The number of BrdU positive cells per 1000 DAPI stained cells in the periglomerular region of OR256-17, OR18-2 and OR37 glomeruli in mean ± SD with significantly lower values for OR37 glomeruli with *p* = 0.0023 compared to OR256-17 and *p* = 0.0256 compared to OR18-2 glomeruli. n.s. *p* > 0.05; *p < 0.05; **p < 0.01.

**Figure 3:**
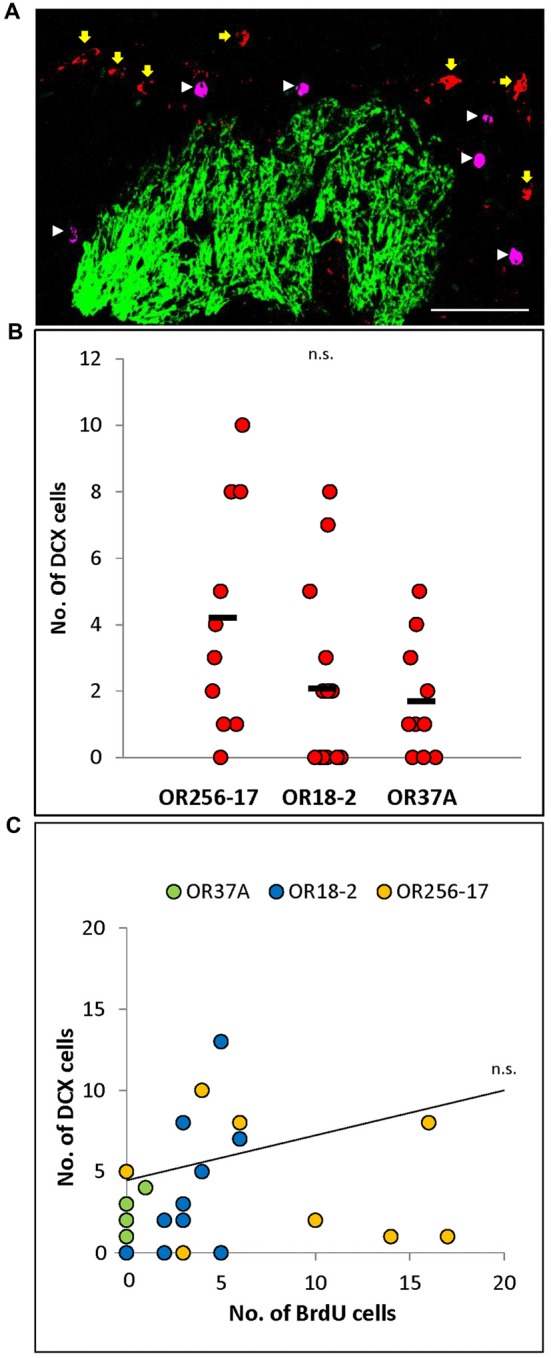
Doublecortin positive cells in the periglomerular region of specific glomeruli. **(A)** Exemplary confocal image of BrdU (magenta, white arrowheads) and doublecortin (red, yellow arrows) double staining around an OR256-17 glomerulus (green). No co-labeling of cells was observed. Scale bar: 50 μm.** (B)** Scatter plot depiction of values for individual glomeruli. The values obtained for individual glomeruli vary notably, with a tendency to more doublecortin positive cells around OR256-17 glomeruli, fewer around OR18-2 glomeruli and still less doublecortin positive cells around OR37A glomeruli. **(C)** The number of doublecortin positive cells does not correlate with the number of BrdU positive cells at 23 days after BrdU injection for individual glomeruli. n.s. *p* > 0.05.

**Figure 4:**
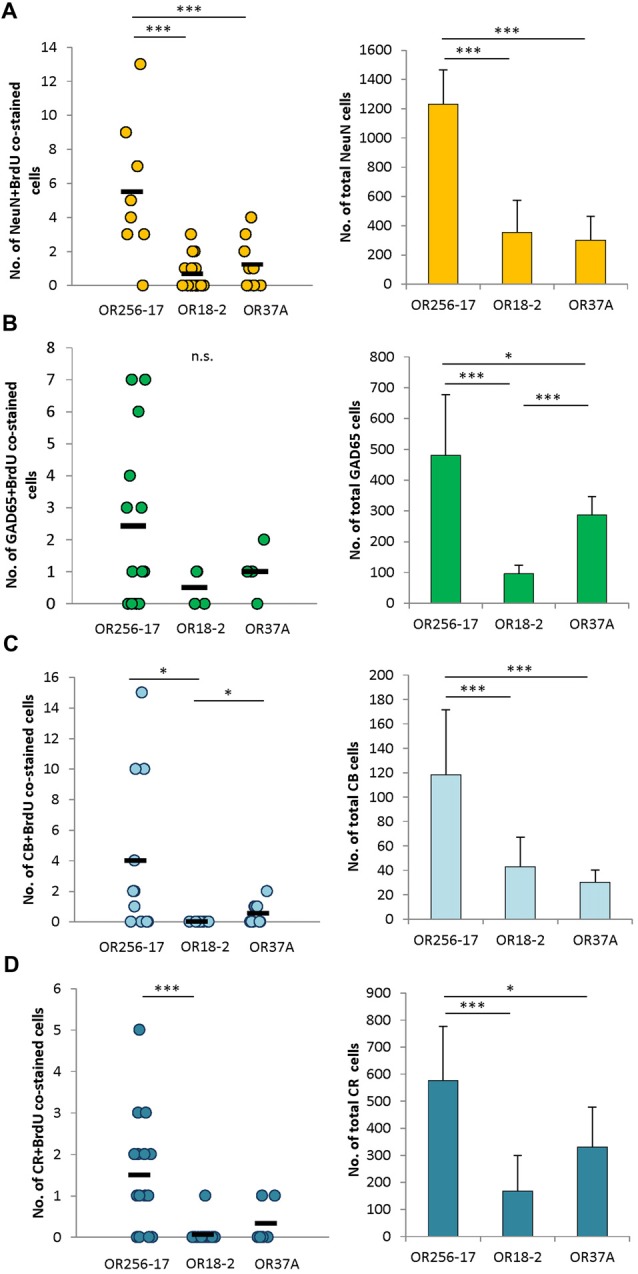
Subtype analyses of adult-born cells in the periglomerular region.** (A)** Scatterplot depicting the total number of NeuN and BrdU double stained cells around individual glomeruli of OR256-17, OR18-2 and OR37A, respectively. Black boxes represent mean values. The total number of NeuN immunoreactive cells in mean ± SD around the different glomerulus types is shown in the bar graph. **(B)** Scatterplot depicting the total number of GAD65 and BrdU double stained cells around individual glomeruli of OR256-17, OR18-2 and OR37A, respectively. Black boxes represent mean values. The total number of GAD65 positive cells in mean ± SD around the different glomerulus types is shown in the bar graph. **(C)** Scatterplot depicting the total number of calbindin and BrdU double stained cells around individual glomeruli of OR256-17, OR18-2 and OR37A, respectively. Black boxes represent mean values. The total number of calbindin immunoreactive cells in mean ± SD around the different glomerulus types is shown in the bar graph. **(D)** Scatterplot depicting the total number of calretinin and BrdU double stained cells around individual glomeruli of OR256-17, OR18-2 and OR37A, respectively. Black boxes represent mean values. The total number of calretinin immunoreactive cells in mean ± SD around the different glomerulus types is shown in the bar graph. n.s. *p* > 0.05; *p < 0.05; ***p < 0.001.

**Table 2:** Number of analyzed individuals.

Experiment	Mouse strain	# of individuals	# of glomeruli
*BrdU single labeling*			
	OR37A-IRES-tau-GFP	3	6
	OR37B-IRES-tau-lacZ	4	9
	OR37C-IRES-tau-GFP	4	9
	OR18-2-IRES-tau-GFP-tau-lacZ	4	10
	OR256-17-IRES-tau-GFP	3	10
*DCX + BrdU co-labeling*			
	OR37A-IRES-tau-lacZ	4	10
	OR18-2-IRES-tau-GFP-tau-lacZ	3	14
	OR256-17-IRES-tau-GFP	3	10
*NeuN + BrdU co-labeling*			
	OR37A-IRES-tau-lacZ	3	9
	OR18-2-IRES-tau-GFP-tau-lacZ	4	18
	OR256-17-IRES-tau-GFP	4	12
*GAD65 + BrdU co-labeling*			
	OR37A-IRES-tau-GFP	4	5
	OR18-2-IRES-tau-GFP-tau-lacZ	4	4
	OR256-17-IRES-tau-GFP	7	14
*TH + BrdU co-labeling*			
	OR37A-IRES-tau-GFP	2	4
	OR18-2-IRES-tau-GFP-tau-lacZ	1	4
	OR256-17-IRES-tau-GFP	1	4
*Calretinin + BrdU co-labeling*			
	OR37A-IRES-tau-GFP	4	6
	OR18-2-IRES-tau-GFP-tau-lacZ	4	17
	OR256-17-IRES-tau-GFP	4	16
*Calbindin + BrdU co-labeling*			
	OR37A-IRES-tau-GFP	3	6
	OR37A-IRES-tau-lacZ	1	3
	OR18-2-IRES-tau-GFP-tau-lacZ	4	11
	OR256-17-IRES-tau-GFP	3	11

*Number of glomeruli, in this study referred to as the number of *n*, and the corresponding number of individuals deployed in the regarding experiment is shown. The number of mice is shown per mouse strain, in which a specific glomerulus is labeled*.

## Results

### BrdU Labeling in the Olfactory Bulb at Different Time Points After Injection

Based on the recent finding that OR37 glomeruli are characterized by a remarkably stable size and a specified periglomerular network, in this study the structural plasticity of neuronal networks around OR specific glomeruli was investigated. Adult born cells in the glomerular layer were visualized by immunostaining of the proliferation marker BrdU. For this approach, adult mice were intraperitoneally injected with BrdU for four consecutive days, three times a day. In initial experiments the optimal time point for the analysis of adult born cells in the olfactory bulb was determined. Tissue sections of the olfactory bulb were analyzed at 12, 19, 23 or 26 days after the last BrdU injection. BrdU positive cells in the olfactory bulb were visualized by immunostainings with a specific antibody against BrdU (Figure 1). In tissue sections from mice which did not receive a previous BrdU injection no positive signals were observed (not shown). In order to determine the optimal time point of analysis, the number of immunostained BrdU positive cells in the granule cell layer was evaluated, since in this part of the bulb the number of adult-born cells is high and the efficacy of the BrdU labeling can be easily estimated. At day 12 after the last BrdU injection, only a small number of BrdU positive cells was observed. The number of labeled cells was slightly higher at day 19 and peaked at day 23 after the last injection. At day 26, the number of BrdU positive cells was already declining (Figure 1A). At day 23, a substantial number of BrdU labeled cells was also visible in the glomerular layer; therefore, this time point was chosen for further analyses of OR specific glomeruli (Figure 1B).

### BrdU Positive Cells in the Vicinity of Odorant Receptor Specific Glomeruli

To investigate adult born cells around the specific glomeruli for different ORs, transgenic mice were used in which the glomeruli for distinct OR types are fluorescently labeled. Mice were injected with BrdU as described above and analyzed 23 days after the last injection. Tissue sections of the olfactory bulbs were immunostained for BrdU (Figure 2A). To consider the different size of individual glomeruli, the number of BrdU labeled cells was related to the number of DAPI stained cells in the proximal vicinity of labeled glomeruli, as described in a previous study (Maier et al., 2017). The number of BrdU labeled cells per 1000 DAPI stained cells was determined around the respective glomeruli. For glomeruli of OR18-2, an average of 4.3 ± 1.5 BrdU positive cells per 1000 DAPI cells was determined (*n* = 10) and a similar value of 4.9 ± 1.3 per 1000 cells for OR256-17 glomeruli (*n* = 10; Figure 2B). For all three OR37 receptor subtypes, much lower numbers were obtained. Moreover, the numbers were highly similar for the three subtypes with 1.6 ± 0.8 BrdU positive cells for OR37A glomeruli (*n* = 6), 1.6 ± 0.7 for OR37B (*n* = 17) and 1.7 ± 0.7 for OR37C glomeruli (*n* = 9; Figure 2B). These data indicate significantly less BrdU labeled cells in the vicinity of OR37 glomeruli than around OR256-17 (*p* = 0.0023) or OR18-2 glomeruli (*p* = 0.0256). This result might imply that in the periglomerular network of OR37 glomeruli, adult born neurons are added at a lower rate compared to glomeruli of other OR types. For further analyses, the OR37A glomerulus was chosen as a representative for OR37 glomeruli.

### Doublecortin Positive Cells in the Periglomerular Region

BrdU labeled cells in the glomerular layer at day 23 after the injection are presumably beyond an immature neuroblast stage but rather in the maturation process towards periglomerular neurons (Belluzzi et al., 2003; Whitman and Greer, 2007). To assess this notion, double staining experiments on tissue sections derived from mice 23 days after BrdU injection were performed, labeling both, BrdU and doublecortin, which is a well-established marker for migrating neuroblasts (Brown et al., 2003; Karl et al., 2005). In none of the experiments, any co-localization of BrdU and doublecortin signals was observed in the periglomerular region of the three glomerulus types as shown exemplarily in Figure 3A. This result indicates that at the time point of analysis the BrdU labeled cells do not express doublecortin, supporting the hypothesis that by these approaches, independent subpopulations of adult born cells were labeled.

To evaluate whether the low number of BrdU labeled cells in the periglomerular area of OR37 glomeruli may be a consequence of a lower number of neuroblasts which arrive at these glomeruli, doublecortin positive cells in the periglomerular region of the three different glomerulus types were quantified. The results indicate that the number of doublecortin cells was highly variable for individual glomeruli as depicted in the scatterplot in Figure 3B. For OR256-17 glomeruli, numbers ranged from 0 to 10 cells per glomerulus (*n* = 10). For glomeruli of OR18-2, numbers ranged from 0 to 8 cells per glomerulus (*n* = 14). For the OR37A glomeruli, the numbers were even lower, ranging from 0 to 5 doublecortin labeled cells per glomerulus (*n* = 10). Overall, the tendency that less doublecortin positive cells were present in the vicinity of OR37A glomeruli is in line with the results of the BrdU experiments. However, the numbers for individual glomeruli obtained in the co-labeling experiments for these two markers did not show any correlation (Figure 3C). This might be a consequence of the highly variable number of doublecortin positive cells and might indicate that the number of more mature adult born neurons labeled by BrdU was independent from the number of younger neuroblasts labeled by doublecortin for individual glomeruli.

### Subtype Analyses of Adult Born Cells in the Periglomerular Region

Towards a further characterization of the adult born cells around receptor specific glomeruli, attempts were made to identify the molecular phenotype of these cells. Therefore, tissue sections were probed for a co-staining of BrdU and several markers for neurons in the periglomerular area. The absolute numbers obtained for co-stained cells are depicted as scatterplots in Figure 4. Immunhistochemical co-stainings of BrdU with NeuN, a marker for mature neurons (Mullen et al., 1992), resulted in 0–13 cells around OR256-17 glomeruli (*n* = 12), 0–3 cells for OR18-2 glomeruli (*n* = 18) and 0–4 co-stained cells for OR37A glomeruli (*n* = 9; Figure 4A). The total number of NeuN labeled cells around the different glomeruli is shown in the bar graph. The distribution of NeuN and BrdU co-stained cells roughly follows the distribution pattern of the total NeuN population, with the highest value for OR256-17 (1230 ± 237.07) and considerably lower numbers for OR18-2 (354.72 ± 220.74) and OR37A (300.89 ± 165.01). These results suggest that a similar fraction of adult born cells develops towards a NeuN-positive neuronal fate.

The majority of neurons in the periglomerular region are presumably GABAergic neurons (Panzanelli et al., 2007; Parrish-Aungst et al., 2007). To identify adult born GABAergic cells around individual glomeruli, transgenic mice were used in which neurons expressing the glutamate decarboxylase GAD65 also express the red fluorescent protein tdTomato. Determining the number of BrdU positive cells which were also labeled by GAD65-tdTomato resulted in 0–7 co-stained cells around OR256-17 glomeruli (*n* = 14) and low numbers for OR18-2 glomeruli with only 0–1 cell (*n* = 4) and for OR37A glomeruli, where 0–2 cells were observed (*n* = 5; Figure 4B). The number of double stained cells hereby seems to reflect the total number of GAD65 cells, with the highest number around OR256-17 glomeruli (480.9 ± 196.6), the lowest numbers for OR18-2 glomeruli (95.6 ± 28.4) and again slightly higher for OR37A (287.6 ± 58.7). These results suggest a similar portion of adult born cells differentiating towards GAD65-expressing GABAergic neurons. Attempts to identify BrdU labeled cells which also express tyrosine hydroxylase (TH) by a specific antibody did not result in any co-labeling visible (data not shown).

In a previous study, we have shown that between OR37A glomeruli vs. OR256-17 and OR18-2 glomeruli the subpopulations of periglomerular cells which are characterized by distinct calcium binding proteins, such as calbindin and calretinin, varied significantly (Maier et al., 2017). Determining the number of cells co-labeled for BrdU and calbindin revealed a low number of co-stained cells (0–2) for the OR37A glomeruli (*n* = 9), compared to 0–15 cells for OR256-17 (*n* = 11; Figure 4C). In the vicinity of OR18-2 glomeruli, no double stained cells were found (*n* = 11). Regarding the total number of calbindin expressing cells around the different glomeruli, for OR37A, a relatively low number of 30.3 ± 10.1 cells was found, compared to 43 ± 24.1 for OR18-2 and 118.6 ± 53 for OR256-17.

For calretinin and BrdU, the number of co-labeled cells was generally quite low with 0–5 co-stained cells around OR256-17 glomeruli (*n* = 16). Only one co-labeled cell was observed in total for OR18-2 glomeruli (*n* = 17) and similarly for OR37A glomeruli where only two double stained cells were observed (*n* = 6; Figure 4D). This finding is of particular interest, as OR37A glomeruli harbor a relatively high number of calretinin expressing cells with 330.3 ± 148.7 compared to 167.7 ± 132.4 for OR18-2 glomeruli. For OR256-17, a number of 576.5 ± 200.6 calretinin positive cells was observed. Taken together, the cell populations expressing the calcium binding proteins calbindin or calretinin comprised only few adult born cells overall which might indicate a low rate of neuronal turnover. Together, the results of this study indicate a relatively low proportion of BrdU labeled adult born cells in the vicinity of OR37 glomeruli, as well as a tendency to a lower number of immature doublecortin labeled neuroblasts around OR37A glomeruli. These observations may be indicative for a particularly stable neuronal network around OR37 glomeruli.

## Discussion

In this study, the structural dynamic of periglomerular cells in the vicinity of OR37 glomeruli was investigated in comparison to glomeruli of other OR types. In a number of recent studies it has been demonstrated that the structural features of glomeruli and their surroundings are quite variable (Weruaga et al., 2001; Kobayakawa et al., 2007; Luo, 2008; Zapiec and Mombaerts, 2015). The recent finding that OR37 glomeruli are particularly stable in size and possess a rather distinct periglomerular network (Maier et al., 2017) has led to the hypothesis that the network of periglomerular cells of the OR37 glomeruli might be more stable than for other glomeruli, which might be reflected by a lower rate of cell replacement. In fact, 23 days after BrdU injection, less BrdU positive cells were found in the surrounding of OR37 glomeruli compared to glomeruli of other receptor types. The generally low numbers of adult born cells in the periglomerular region of individual glomeruli can be explained by the fact that only a small portion of adult born interneurons reach the Glomerular Layer (Hack et al., 2005; Alonso et al., 2008; García-González et al., 2016; Díaz et al., 2017), so that, under unstimulated conditions, only few cells can be observed in the direct surrounding of a single glomerulus (Khodosevich et al., 2013). In view of the fact that mice were injected with BrdU at four consecutive days three times a day, the data indicate a lower number of newly generated periglomerular cells which were born up to 27 days before analyses. The maturational stage of BrdU positive cells was assessed by double labeling experiments with an antibody for doublecortin, which is considered a marker for neuroblasts (Brown et al., 2003). The finding that no cells were co-stained for BrdU and DCX at the time point of analyses suggests that BrdU labeled cells were beyond a stage detectable by DCX labeling and thus cannot be considered as neuroblasts; this observation is in line with the findings of previous studies (Belluzzi et al., 2003; Whitman and Greer, 2007; García-González et al., 2016; Langenfurth et al., 2016). The notion that a low number of BrdU positive cells in the vicinity of OR37 glomeruli might reflect a generally low number of neuroblasts reach the OR37-area in the antero-ventral region of the bulb was supported by the finding that also less DCX stained cells were found for OR37A glomeruli compared to OR18-2 and OR256-17 glomeruli.

It has been previously proposed that the various periglomerular cell populations might contribute unequally to the adult plasticity processes of the network (Bovetti et al., 2009; Sawada et al., 2011; Díaz et al., 2017). In the attempts to determine the molecular phenotype of BrdU labeled cells around the distinct glomerulus types, double staining experiments for BrdU and the neuronal marker NeuN were performed. A generally low number of adult born periglomerular cell types in the glomerular area was also reported in a previous study (Díaz et al., 2017). Therefore, the numbers of double labeled cells are given in absolute numbers, as well as the total numbers of marker labeled populations towards a direct comparison. A detailed description of relative numbers of different PGC populations in dependence of glomerulus size are given in a previous study, indicating glomerulus-specific cell numbers for CB and CR, but not NeuN nor GAD65 (Maier et al., 2017). The finding that the number of co-stained cells was proportional to the total number of NeuN positive cells at the different glomeruli indicates that also a similar fraction of adult born cells is in the process towards a neuronal fate characterized by NeuN. A similar result was obtained by double staining with BrdU and GAD65, a marker for GABAergic neurons (Erlander et al., 1991; Besser et al., 2015); the number of double stained cells was proportional to the total number of GAD65 positive cells for the different glomeruli. Dopaminergic neurons, characterized by the expression of TH, are frequently discussed in the context of adult neurogenesis in the OB (Lledo et al., 2006; García-González et al., 2016; Yoshihara et al., 2016), however, none of the BrdU labeled cells was found to be stained for TH. This may be due to the fact that adult born TH-positive cells in the glomerular layer apparently require a longer time span than other cell types to reach a mature stage (Winner et al., 2002). The number of periglomerular cells which are distinguished based on the calcium binding proteins calbindin and calretinin varied significantly for different glomerulus types with a comparably high number at the OR37A glomeruli (Maier et al., 2017). An assessment of BrdU-positive cells for either calbindin or calretinin revealed that in this case, the number of double stained cells was not proportional to the total number of marker positive cells. Notably, in spite of the relatively high number of calretinin positive cells at the OR37A glomeruli, only very few BrdU cells were found to be stained for calretinin. This observation might reflect that the turnover rate of the various cell types may be different for the OR specific glomeruli. In conclusion, the results indicate that glomeruli of the OR37 subsystem harbor significantly less adult born cells in their proximity than other glomeruli; an observation which might be indicative for a less dynamic periglomerular network for OR37 glomeruli. This study thereby provides evidence that OR37-related bulbar networks dedicated to social information processing might differ from others in regards to plasticity properties of its components.

## Author Contributions

A-MM, HB and JS conceived and designed the experiments. A-MM performed the experiments, analyzed the data and prepared the figures. A-MM and HB wrote the manuscript. All authors approved the final version of the manuscript.

## Conflict of Interest Statement

The authors declare that the research was conducted in the absence of any commercial or financial relationships that could be construed as a potential conflict of interest.
